# Supplementary material for: Water Quality and Total Microbial Load: A Double-Threshold Identification Procedure Intended for Space Applications
Source: Front Microbiol. 2018 Dec 6;9:2903. doi: 10.3389/fmicb.2018.02903 (PMC6291452; doi:10.3389/fmicb.2018.02903)
Supplement: Supplementary file 1 [file Table_1.DOCX]

**Table S1**. Complete dataset of the water microbial load. Heterotrophic Plate Counts (HPC) were assessed from different cultivation media (i.e., YEA, R2A) and incubation times (i.e., 3 and 7 days). Cultivation-independent techniques with current and promising perspectives for space applications were selected (i.e., ATP-metry, qPCR, flow cytometry - FCM). Samples included chlorinated Tap Waters (cTW1-7), unchlorinated Tap Waters (uTW1-7), Ground Waters (GW1-7), Rivers Waters (RW1-7), and Waste Waters (WW1-7).

|  | **Cultivation based techniques** | | | **Cultivation independent techniques** | | | |
| --- | --- | --- | --- | --- | --- | --- | --- |
|  | **HPC** YEA-3d  CFU/ml | **HPC** YEA-7d  CFU/ml | **HPC** R2A-7d  CFU/ml | **ATP-metry**  pg/ml | **qPCR** 16S  rDNA copies | **FCM** Total  cells/ml | **FCM** Intact  cells/ml |
| **cTW1** | 1.00E+00 | 2.00E+00 | 4.00E+00 | 1.48E-03 | 4.21E+02 | 2.55E+04 | 2.23E+04 |
| **cTW2** | 1.00E+00 | 1.80E+01 | 2.80E+01 | 2.41E-02 | 1.46E+03 | 4.65E+04 | 2.93E+04 |
| **cTW3** | 1.00E+00 | 1.00E+00 | 4.00E+00 | 2.44E-03 | 2.23E+02 | 2.14E+04 | 1.92E+04 |
| **cTW4** | 1.00E+00 | 0.00E+00 | 1.00E+00 | 8.02E-03 | 3.70E+02 | 1.50E+04 | 1.30E+04 |
| **cTW5** | 0.00E+00 | 0.00E+00 | 3.00E+00 | 3.89E-03 | 5.00E+03 | 5.71E+04 | 5.10E+04 |
| **cTW6** | 1.00E+00 | 4.00E+00 | 1.20E+01 | 3.13E-03 | 2.68E+02 | 1.57E+04 | 1.52E+04 |
| **cTW7** | 1.00E+00 | 3.00E+00 | 5.00E+00 | 2.46E-03 | 3.70E+02 | 1.79E+04 | 1.55E+04 |
| **uTW1** | 0.00E+00 | 1.25E+02 | 4.00E+02 | 1.95E+00 | 1.36E+04 | 1.12E+05 | 8.84E+04 |
| **uTW2** | 2.00E+00 | 4.90E+01 | 3.00E+02 | 2.11E+00 | 1.54E+04 | 1.18E+05 | 8.49E+04 |
| **uTW3** | 0.00E+00 | 1.83E+02 | 1.20E+03 | 2.75E+00 | 1.76E+04 | 1.45E+05 | 1.03E+05 |
| **uTW4** | 1.00E+00 | 3.00E+02 | 3.00E+02 | 2.09E+00 | 1.87E+04 | 1.28E+05 | 1.02E+05 |
| **uTW5** | 1.00E+00 | 3.00E+02 | 3.00E+02 | 1.55E+00 | 9.39E+03 | 1.19E+05 | 6.93E+04 |
| **uTW6** | 0.00E+00 | 2.30E+01 | 5.40E+02 | 1.41E+00 | 8.85E+03 | 1.25E+05 | 8.94E+04 |
| **uTW7** | 0.00E+00 | 1.20E+01 | 3.00E+02 | 1.57E+00 | 1.05E+04 | 1.30E+05 | 8.11E+04 |
| **GW1** | 1.00E+00 | 1.00E+00 | 3.00E+02 | 2.18E-01 | 8.64E+03 | 4.61E+04 | 4.37E+04 |
| **GW2** | 0.00E+00 | 2.00E+00 | 2.00E+02 | 1.67E-01 | 1.14E+04 | 2.92E+04 | 2.78E+04 |
| **GW3** | 0.00E+00 | 0.00E+00 | 2.00E+02 | 7.65E-01 | 1.89E+04 | 6.39E+04 | 6.17E+04 |
| **GW4** | 7.00E+00 | 1.00E+01 | 2.00E+01 | 1.95E-01 | 5.69E+03 | 2.48E+04 | 2.37E+04 |
| **GW5** | 0.00E+00 | 1.00E+00 | 6.00E+00 | 1.48E+00 | 1.18E+04 | 1.41E+04 | 1.28E+04 |
| **GW6** | 0.00E+00 | 0.00E+00 | 1.20E+01 | 2.00E+00 | 1.71E+04 | 1.19E+04 | 1.01E+04 |
| **GW7** | 0.00E+00 | 0.00E+00 | 7.30E+01 | 1.43E+01 | 8.88E+04 | 3.42E+04 | 2.92E+04 |
| **RW1** | 8.30E+01 | 1.53E+02 | 7.00E+02 | 3.41E+00 | 3.34E+04 | 3.43E+04 | 2.73E+04 |
| **RW2** | 7.20E+02 | 8.50E+02 | 6.50E+03 | 2.92E+01 | 1.39E+05 | 1.44E+05 | 1.26E+05 |
| **RW3** | 4.50E+01 | 4.90E+01 | 3.00E+02 | 8.66E-01 | 8.67E+03 | 2.09E+04 | 1.93E+04 |
| **RW4** | 4.90E+02 | 6.20E+02 | 1.00E+04 | 3.60E+02 | 1.50E+06 | 1.42E+06 | 1.01E+06 |
| **RW5** | 1.88E+03 | 1.96E+03 | 2.94E+03 | 3.96E+00 | 2.21E+04 | 3.23E+04 | 2.92E+04 |
| **RW6** | 5.00E+02 | 5.90E+02 | 1.20E+04 | 1.58E+02 | 7.49E+05 | 4.32E+05 | 3.84E+05 |
| **RW7** | 1.33E+03 | 1.51E+03 | 1.00E+04 | 5.60E+01 | 7.99E+05 | 5.98E+05 | 4.62E+05 |
| **WW1** | 1.00E+02 | 3.00E+02 | 3.00E+04 | 1.94E+02 | 2.72E+06 | 2.07E+06 | 1.86E+06 |
| **WW2** | 3.00E+04 | 3.00E+04 | 3.00E+06 | 5.90E+03 | 9.81E+07 | 2.92E+07 | 2.56E+07 |
| **WW3** | 3.10E+03 | 4.70E+03 | 4.00E+04 | 2.80E+02 | 6.89E+06 | 6.62E+06 | 5.63E+06 |
| **WW4** | 1.92E+04 | 3.00E+04 | 5.00E+04 | 2.77E+03 | 7.23E+07 | 1.04E+06 | 9.31E+05 |
| **WW5** | 3.00E+04 | 3.00E+04 | 3.00E+06 | 2.72E+03 | 9.15E+06 | 1.51E+07 | 1.20E+07 |
| **WW6** | 1.00E+02 | 2.00E+02 | 1.00E+04 | 2.09E+02 | 2.33E+06 | 1.32E+06 | 1.28E+06 |
| **WW7** | 4.50E+03 | 7.90E+03 | 2.00E+04 | 3.55E+02 | 3.78E+06 | 2.85E+06 | 2.67E+06 |
